# Sperm Associated Antigen 6 (SPAG6) Regulates Fibroblast Cell Growth, Morphology, Migration and Ciliogenesis

**DOI:** 10.1038/srep16506

**Published:** 2015-11-20

**Authors:** Wei Li, Abir Mukherjee, Jinhua Wu, Ling Zhang, Maria E. Teves, Hongfei Li, Shanti Nambiar, Scott C. Henderson, Alan R. Horwitz, Jerome F. Strauss III, Xianjun Fang, Zhibing Zhang

**Affiliations:** 1 Department of Obstetrics & Gynecology, Virginia Commonwealth University, Richmond, VA, 23298; 2 Department of Biochemistry and Molecular Biology, Virginia Commonwealth University, Richmond, VA, 23298; 3 School of Public Health, Wuhan University of Science and Technology, Wuhan, Hubei 430065, China; 4 Department of Anatomy and Neurobiology, Virginia Commonwealth University, Richmond, VA, 23298; 5 Department of Cell Biology, University of Virginia, Charlottesville, VA 22908, USA

## Abstract

Mammalian *Spag6* is the orthologue of Chlamydomonas *PF16*, which encodes a protein localized in the axoneme central apparatus, and regulates flagella/cilia motility. Most *Spag6*-deficient mice are smaller in size than their littermates. Because SPAG6 decorates microtubules, we hypothesized that SPAG6 has other roles related to microtubule function besides regulating flagellar/cilia motility. Mouse embryonic fibroblasts (MEFs) were isolated from *Spag6*-deficient and wild-type embryos for these studies. Both primary and immortalized *Spag6*-deficient MEFs proliferated at a much slower rate than the wild-type MEFs, and they had a larger surface area. Re-expression of SPAG6 in the *Spag6*-deficient MEFs rescued the abnormal cell morphology. *Spag6*-deficient MEFs were less motile than wild-type MEFs, as shown by both chemotactic analysis and wound-healing assays. *Spag6*-deficient MEFs also showed reduced adhesion associated with a non-polarized F-actin distribution. Multiple centrosomes were observed in the *Spag6*-deficient MEF cultures. The percentage of cells with primary cilia was significantly reduced compared to the wild-type MEFs, and some *Spag6*-deficient MEFs developed multiple cilia. Furthermore, SPAG6 selectively increased expression of acetylated tubulin, a microtubule stability marker. The *Spag6*-deficient MEFs were more sensitive to paclitaxel, a microtubule stabilizer. Our studies reveal new roles for SPAG6 in modulation of cell morphology, proliferation, migration, and ciliogenesis.

Mammalian Sperm associated antigen 6 (SPAG6) is the orthologue of the green algae model organism, *Chlamydomonas reinhardtii* Paralyzed flagella 16 (*PF16*)^1^,^2^. *PF16* encodes a protein localized to the central apparatus of the “9 + 2” axoneme^3^. In addition to mouse and *Chlamydomonas*, *Spag6/PF16* orthologus have been identified in several other species, including trypanosomes, Plasmodium, Giardia, frogs, chickens, and turtles^1^,^2^,^3^,^4^,^5^,^6^,^7^. In most of these organisms, the encoded protein has a similar role in regulation of cilia/flagella motility as described in *Chlamydomonas*^1^,^2^,^3^.

Recent findings suggest that besides regulating flagellar/ciliary motility, SPAG6 has other functions *in vivo* that had not been previously recognized from the studies of model organisms like *Chlamydomonas*. For example, silencing the *Spag6* gene in *Xenopus* larvae leads to disruption of orientation of the basal bodies^7^. Several other studies demonstrated that *SPAG6* gene expression is up-regulated in some primary cancers and cancer cell lines^8^,^9^,^10^, and silencing *SPAG6* expression by SPAG6-short hairpin RNA (shRNA) lentivirus dramatically inhibited tumor growth and increased apoptosis *in vivo*^11^.

Diverse functions of SPAG6 have been discovered in mice in which the *Spag6* gene was disrupted^12^. The majority of *Spag6*-deficient mice died from hydrocephalus before adulthood, and the surviving males were infertile due to reduced ciliary/flagellar motility^12^. Even though an abnormal axoneme ultrastructure was discovered in the *Spag6*-deficient sperm^12^, the cilia present in brain ependymal epithelial cells and trachea epithelial cells from the mutant mice contained “9 + 2” axonemes that appeared to be grossly intact^13^. However, we found a ciliogenesis defect in the brain ependymal, trachea and middle ear epithelial cells. Axoneme/basal feet orientation, and tracheal epithelial cell polarity were altered and associated with disrupted distribution of microtubules^14^. We concluded that hydrocephalus in the *Spag6*-deficient mice might be largely due to disrupted polarity in the epithelial cells. More recent studies demonstrated a role for *Spag6* in the pathogenesis of otitis media in mice, possibly by regulation of cilia/basal body polarity through the planar cellular polarity (PCP)-dependent mechanisms in the middle ear and Eustachian tubes^15^.

Almost all the *Spag6*-deficient mice were smaller in size compared to their wild-type littermates. However, the mechanism for the growth stunting was poorly understood. We previously showed that SPAG6 associates with microtubules in transfected CHO cells^16^,^17^. Therefore, we hypothesized that besides regulating ciliary/flagellar motility, SPAG6 exerts an effect on the functions of microtubules. To further explore the mechanisms accounting for the unexpected phenotypes in the *Spag6*-deficient mice, we took advantage of MEFs from *Spag6*-deficient embryos and their wild-type littermates. Surprisingly, these *Spag6* null MEFs showed a markedly different morphology from wild-type MEFs. Specifically, they were generally larger in size. Many cells exhibited larger nuclei and contained multiple cytoplasmic vesicles. The abnormal cellular morphology of *Spag6*-deficient MEFs could be reversed by re-expression of SPAG6. The *Spag6*-deficient MEFs grew at a much slower rate. The motility of the cells was also dramatically reduced. Furthermore, *Spag6*-deficient MEFs showed defects in adhesion, cell division and formation of primary cilia. Finally, SPAG6 expression was associated with increased levels of acetylated tubulin, and *Spag6*-deficient MEFs were more sensitive to paclitaxel, a microtubule stabilizer. These findings suggest a broad range of cellular functions of SPAG6 in proliferation, migration, adhesion, cell division, and ciliogenesis. These effects are possibly mediated by alterations in tubulin acetylation and/or microtubule stability.

## Materials and Methods

### *Spag6* mutant mice

*Spag6* mutant mice were previously generated in our laboratory^12^. All animal work was approved by Virginia Commonwealth University’s Institutional Animal Care & Use Committee (protocol AD10000167) in accordance with Federal and local regulations regarding the use of non-primate vertebrates in scientific research.

### Plasmid constructs

Mouse *Spag6* full-length complementary deoxyribonucleic acid (cDNA) was amplified from a mouse testis cDNA using the following primer set: forward: 5′-GGATCCATGAGCCAGCGGCAGGTGCTGC-3′ (BamH1), and reverse: 5′-GTCGACGTTCACAGTGGTTGGTAGCTGTC-3′(SalI), after sequencing, the *Spag6* cDNA was cloned into BamH1/SalI sites of pTarget. *Spag*6/pTarget plasmid was digested by BamH1/NotI, and the released *Spag6* fragment was ligated to BamH1/NotI sites of pcDNA3 vector, creating pcDNA3-*Spag6* plasmid.

### Mouse embryonic fibroblasts

Mouse embryonic fibroblasts (MEFs) were isolated from E13.5 embryos as described^18^. After removing head and heart tissues, the remaining embryo were minced with scissors and digested with 0.25% trypsin/EDTA at 37 °C for 15 min. After digestion and removal of undigested tissues, the cells were spun briefly, plated into a 10-cm dish, and allowed to grow to subconfluence in DMEM containing 10% FBS. The cells were then either frozen as passage 1 or subcultured at a 1:4 ratio for experiments. Immortalized cell lines were established from primary MEFs using standard protocols^19^.

### Other cells and transfection

COS-1 and CHO cells were cultured in DMEM or DMEM/F12 respectively, supplemented with 10% FBS, 100 U/ml penicillin, and 100 μg/ml streptomycin. Transfection was performed using X-tremeGENE Transfection Reagents from Roche following the procedure recommended by the company.

### Cell surface area measurements

The MEFs were cultured in chambered slides. The second day after seeding, the cells were stained with crystal violet, and cell surface area was measured using Image J software.

### Cell proliferation assay

MEFs of the same passages were seeded at a density of 5 × 10^3^ cells/well into 24-well plates. The cell numbers were determined with a Coulter counter on daily basis for 5 days. Cell growth curves were generated to compare growth rates of cells of different genotypes.

### Cell migration assay

Cell migration was measured using Transwell chambers (pore size 8 μm; BD Biosciences, San Jose, CA; cat. no. 354578). Transwells were coated with 10 μg/ml collagen 1 and placed in the lower chamber containing DMEM with 10% FBS. Cells suspended in serum-free DMEM were added to the upper chamber at 2.5 × 10^4^ cells/well. Cells were allowed to migrate for 6 h at 37 °C. Nonmigrated cells were removed from the top filter surface with a cotton swab. Migrated cells attached to the underside of the Transwells were washed with PBS and stained with crystal violet and counted under a microscope.

### Scratch wound-healing assay

MEFs were seeded in 8 chamber slides and cultured until a monolayer was formed. A straight line “scratch” was created with a P2 pipet tip. Cell debris was removed by washing the cells once with PBS and then replace with normal cell culture medium and time-lapse images were taken for 12 hours, using a Zeiss AxioObserver microscope equipped with a stage incubator to regulate temperature, humidity and CO_2_ levels. The distance between the two migrating front lines was measured after recording the videos, as Distance 1 with Distance 0 at the initiation of the scratch experiment. The migration rate was calculated as: Distance 1/Distance 0, with the greater value indicating a faster migration rate.

### Cell adhesion and spreading assay

To compare cell attachment of wild-type and *Spag6*-deficient MEFs, the same number of cells were seeded into 6-well plates. After the cells settled down for 20, 40 and 60 minutes, the cells were washed with PBS, and the attached cells were trpsinized and the cell numbers were counted, and the ratio of attached cells/total cells was calculated.

To observe F-actin distribution during cell adhesion, once the cells were seeded after the indicated time, the cells were stained with 488-labeled phalloidin.

### Scanning Electron Microscopy (SEM)

For SEM analysis, MEFs were cultured on glass cover slides and were processed by standard methods^20^. Images were taken with a Zeiss EVO 50 XVP SEM at Virginia Commonwealth University’s Microscopy Facility.

### Western blotting

Mouse tissues or cells were lysed in radioimmunoprecipitation assay (RIPA) buffer and protein concentration was measured. Total proteins were resolved by SDS-PAGE, transferred to Immuno-Blot membrane [poly(vinylidene difluoride)]; Bio-Rad, Hercules, CA), and immunoblotted with indicated antibodies. Immunocomplexes were visualized with an enhanced chemiluminescence detection kit (Amersham, Piscataway, NJ), using horseradish peroxidase–conjugated secondary antibodies (Cell Signaling).

### Co-immunoprecipitation assay

COS-1 cells were transfected with SPAG6/pTarget plasmid. Forty-eight hours later, the cells were collected for co-immunoprecipitation assay using the protocol described previously^21^. Briefly, the supernatants were then incubated with preimmune serum (negative control) or an anti-SPAG6 polyclonal antibody at 4 °C for 2 h, and protein A beads were added with a further incubation at 4 °C overnight. The beads were washed with immune-precipitation buffer three or four times, and loading buffer was then added to the beads, which were boiled at 100 °C for 10 min; the samples were then processed for Western blotting with monoclonal anti-acetylated tubulin antibody.

### Confocal microscopy

Cells were washed with PBS, fixed with 4% formaldehyde/PBS, and permeabilized with 1% Triton X-100 for 5 min at 37 °C; blocked 1 h at room temperature with 10% goat serum in PBS. After the cells were incubated overnight with the indicated primary antibodies at 4 °C in a moist chamber, cells were washed three times with PBS and incubated 1 h with the secondary antibodies. The cells were washed three times in PBS again, mounted with Vectashield with 4′, 6-diamidino-2-phenylindole (DAPI, Vector laboratories Inc, Burlingame, CA), and sealed with a cover slip. Images were captured by confocal laser-scanning microscopy (Leica TCS-SP2 AOBS).

### Adenovirus expressing mouse SPAG6

An adenovirus expressing SPAG6 was made by Vector BioLabs (Cat number: ADV-272926). A GFP marker was included as an indicator for virus infection. The control virus, Ad-CMV-GFP adenovirus, was also purchased form the same company (Cat number: 1060).

### Cell viability to assay

Wild-type and *Spag6*-deficient MEFs were seeded into 24-well plates. After overnight culture, cells were treated with various concentrations of paclitaxel (Sigma, T7191), a compound that stabilizes the microtubule^22^, for 48 hrs. Both floating and adherent cells were harvested and stained with Trypan blue. Cell viabilities were determined with a TC10 Automatic Cell Counter (Bio-Rad). Each experiment was performed in triplicate for at least three times.

## Results

### Enlarged cell size and slower proliferation of *Spag6*-deficient MEFs

**Figure 1:**
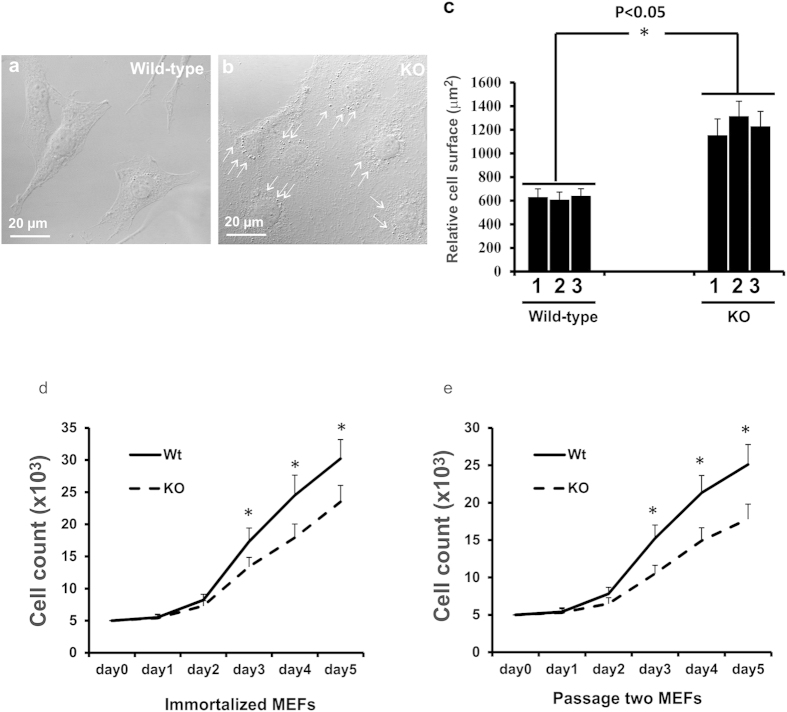
Abnormal cell morphology and proliferation of the *Spag6*-deficient (KO) MEFs. Representative images of MEFs from a wild-type mouse (**a**) and a *Spag6*-deficient mouse (**b**) under a light microscopy. Notice that *Spag6*-deficient MEFs were larger than the wild-type MEFs. There seems to be more vesicles in the mutant cells (arrows in b). Relative cell surface area was measured on MEFs from three wild-type and three *Spag6*-deficient mice, 50 cells were analyzed from each line. The cell surface of *Spag6*-deficient MEFs was significantly greater than that of the wild-type MEFs (**c**). *p < 0.05. Immortalized (**d**) and passage two (**e**) wild-type and *Spag6*-deficient MEFs were seeded in 24 well plates and cell numbers were counted for five continuous days. The data presented are the average from three wild-type and three mutant lines. Notice that the *Spag6*-deficient MEFs grew at a much slower rate than the wild-type MEFs in both immortalized and passage two cells.

MEFs were isolated from *Spag6*-deficient embryos and wild-type littermates. Although they were indistinguishable at early passages, wild-type and *Spag6*-deficient MEFs showed different morphologies when they were immortalized after being passaged more than 15 times. All six lines of the wild-type MEFs generated were more uniform in size, showing characteristic fibroblast-like morphology (Fig. 1a). However, all five lines of *Spag6*-deficient cells generated appeared to be much larger than the wild-type MEFs (Fig. 1b). In addition, the cytoplasm of wild-type MEFs was more smooth. In contrast, *Spag6*-deficient MEFs appeared to have more vesicles. The different cell sizes and morphologies between wild-type and *Spag6*-deficient MEFs were confirmed by SEM (Supplemental Figure 1, left panel). To better visualize the cells, the MEFs were stained with crystal violet, which made it easy to distinguish cell boundaries (Supplemental Figure 1, right panel). Measurement of cell surface areas indicated that the difference was statistically significant between wild-type and *Spag6*-deficient MEFs (Fig. 1c).

Compared to the wild-type MEFs, all of the *Spag6*-deficient MEFs appeared to grow slowly. The cell growth rate of MEFs was monitored by counting cell numbers on sequential days after seeding the same number of cells. Consistent with the growth-retardation phenotype observed in the *Spag6* null mice, *Spag6*-deficient MEFs exhibited a reduced growth rate compared to wild-type MEFs in both immortalized (Fig. 1d) and passage two (Fig. 1e) MEFs.

### Reduced motility of *Spag6*-deficient MEFs

**Figure 2:**
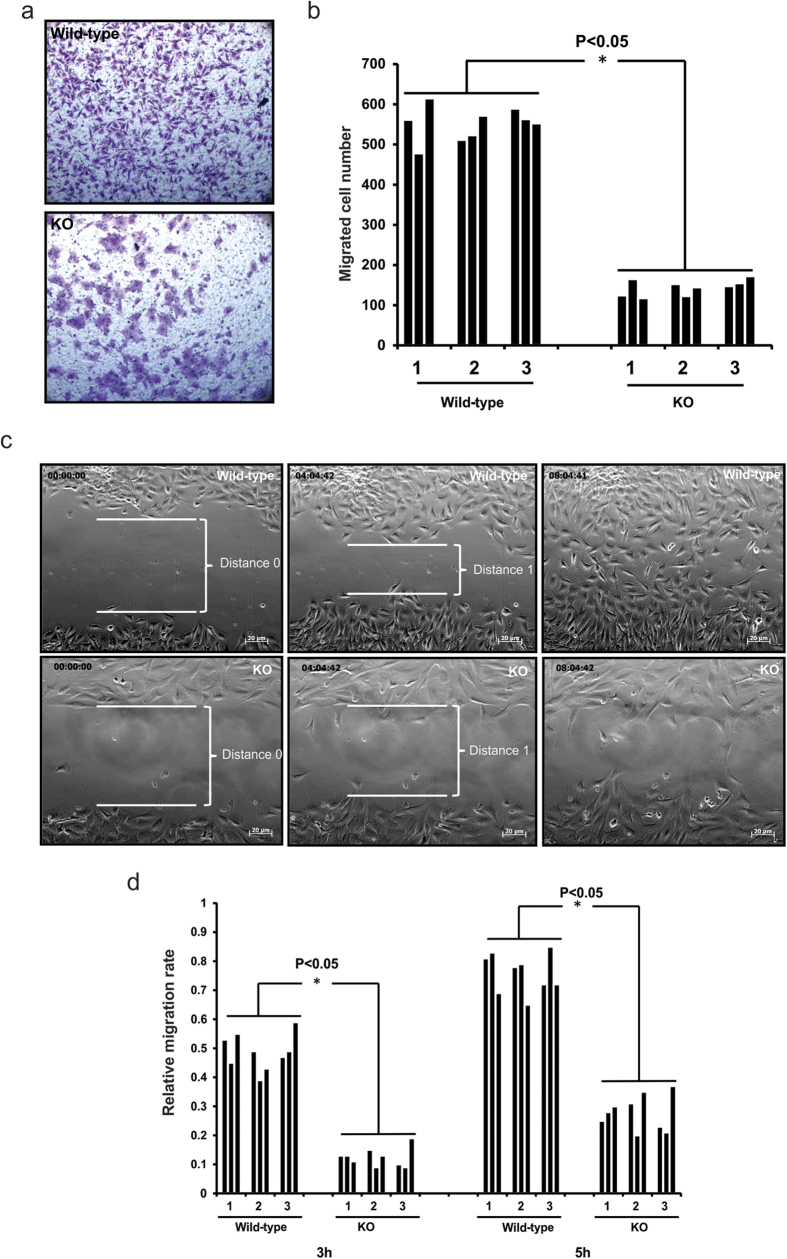
Reduced migration in the *Spag6*-deficient (KO) MEFs. (**a**) Transwell chamber assay. Representative images of the migrated cells on the membranes. Equal numbers of wild-type (upper panel) and *Spag6*-deficient MEFs (lower panel) were loaded, six hours after migration, the membranes were stained with crystal violet. (**b**) The number of migrated cells was counted, and the migrated cell/total cell ratio was calculated. There was a significant reduction in the *Spag6*-deficient MEFs (p < 0.05). Three wild-type and three *Spag6*-deficient cell lines were studied, and each cell line was analyzed three times. (**c**) Wound-healing assay. Representative images after scratch. Upper panel: wild-type MEFs; lower panel: *Spag6*-deficient MEFs. Notice that eight hours after scratch, wild-type MEFs filled the scratch space. However, a significant scratch space was still present in the *Spag6*-deficient MEFs. (**d**) Relative migration rate of the MEFs. The migration distance was measured three and five hours from time 0, and the relative migration rate was calculated as described in the Materials and Methods section. The rate was significantly higher in the wild-type MEFs. *p < 0.05.

Because SPAG6 co-localizes with a subset of microtubules in the transfected cells^12^, we speculated that SPAG6 plays its roles through association and regulation of microtubules^15^. Migration of mammalian cells involves highly coordinated rearrangement of the microtubule cytoskeleton^23^. Thus, we compared migration of wild-type and *Spag6*-deficient MEFs using Transwell chambers. Equal numbers of cells were loaded onto the upper chambers. After six hours of incubation, the cells migrating to the underside of the transwells were quantified by microscopy. As presented in Fig. 2a,b, there was a significantly lower percentage of migrated *Spag6*-deficient cells compared to wild-type MEFs. Consistent with previous observations, *Spag6*-deficient MEFs remained larger following migration.

We further analyzed the motility of these cells using a wound-healing assay where real time movement of cells was monitored by continuous video microscopy for twelve hours. About six hours after a scratch was placed, wild-type MEFs almost completely filled the wound (Fig. 2c, upper panel and Supplemental movie 1). In contrast, there was little decrease in wound width, reflecting limited migration of the *Spag6*-deficient MEFs (Fig. 2c, lower panel, and Supplemental movie 2). The relative migration distance was measured, and the migration rate was calculated, which revealed significant difference between wild-type and *Spag6*-deficient MEFs (Fig. 2d).

### Disrupted distribution of cytoskeleton components during migration and reduced adhesion ability of the *Spag6*-deficient MEFs

**Figure 3:**
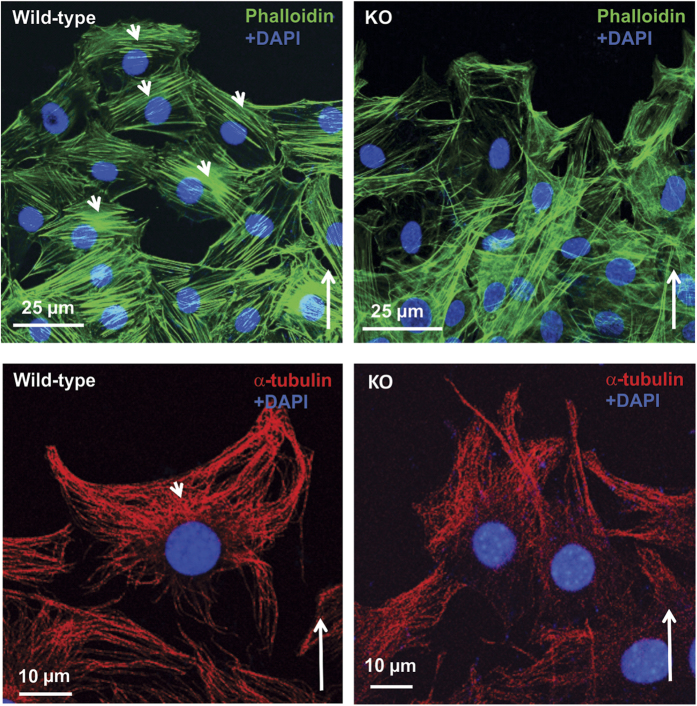
Abnormal distribution of cytoskeleton components in the *Spag6*-deficient (KO) MEFs during migration. Upper panels: F-actin distribution; Lower panels. α–tubulin distribution. Notice that in the wild-type MEFs (left panels), both F-actin and α–tubulin distribute to the migrating front area (arrow heads), but this pattern is lost in the *Spag6*-deficient MEFs. The long arrows show migration direction.

To understand the mechanism underlying the impaired motility in the absence of *Spag6*, we examined the distributions of two major cytoskeleton components, filament actin (F-actin) and α–tubulin in migrating cells. Both F-actin (Fig. 3, upper, left panel) and α–tubulin (Fig. 3, lower, left panel) were distributed near the leading edge of wild-type migrating MEFs. However, in the migrating *Spag6*-deficient MEFs, both F-actin and α–tubulin were present throughout the cytoplasm without a polarized distribution (Fig. 3, upper, right panel for F-actin, and Fig. 3, lower, right panel for α–tubulin).

**Figure 4:**
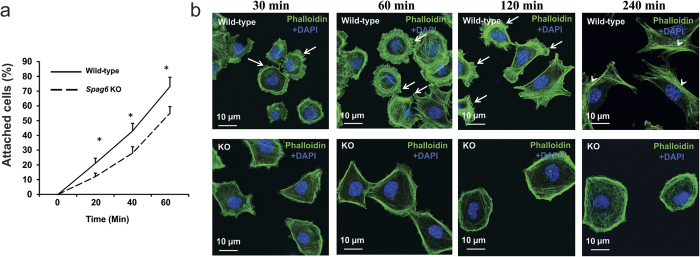
Abnormal adhesion and spreading of in the *Spag6*-deficient (KO) MEFs. (**a**) Percentages of attached cells at different time after seeding. Notice that the percentages of attached cells in the *Spag6*-deficient MEFs are significantly lower than that of the wild-type MEFs at all the three time points analyzed. *p < 0.05. (**b**) Representative images of F-actin distribution during adhesion. The indicated cells were seeded at the indicated time, and subsequently stained with 488-labeled phalloidin. Notice that wild-type MEFs had multiple filopodia during adhesion (arrows). Four hours after adhesion, stress fibers were observed in the wild-type MEFs (arrow heads). However, no obvious filopodia were observed throughout the whole experimental period in the *Spag6*-deficient MEFs. Instead, F-actin was predominately present on the cell surface, and no stress fiber was observed.

Given the disrupted cytoskeleton system during migration in the *Spag6*-deficient MEFs, we hypothesized that the mutant cells also have reduced adhesion ability. We thus compared adhesion behavior between wild-type and *Spag6*-deficient MEFs. Cells firmly attached to the plates were counted at 20 min, 40 min, and 60 min after seeding, the numbers in the *Spag6*-deficient MEFs were significantly lower than in the wild-type MEF cultures (Fig. 4a). The distribution of F-actin was analyzed during cell attachment. Wild-type and *Spag6*-deficient MEFs were seeded into chambered-slides and after the cells had settled for 30, 60, 120 and 240 minutes, they were stained with 488-labeled phalloidin. Wild-type MEFs had multiple filopodia as soon as they attached. Four hours after adhesion, robust stress fibers were observed in the wild-type MEFs. However, no obvious filopodia were observed throughout the whole experimental period (from 30 minutes to four hours) in the *Spag6*-deficient MEFs, F-actin was predominately present on the cell surface, and no stress fibers were observed (Fig. 4b).

### Increased multiple centrosomes in the *Spag6*-deficient MEFs

**Figure 5:**
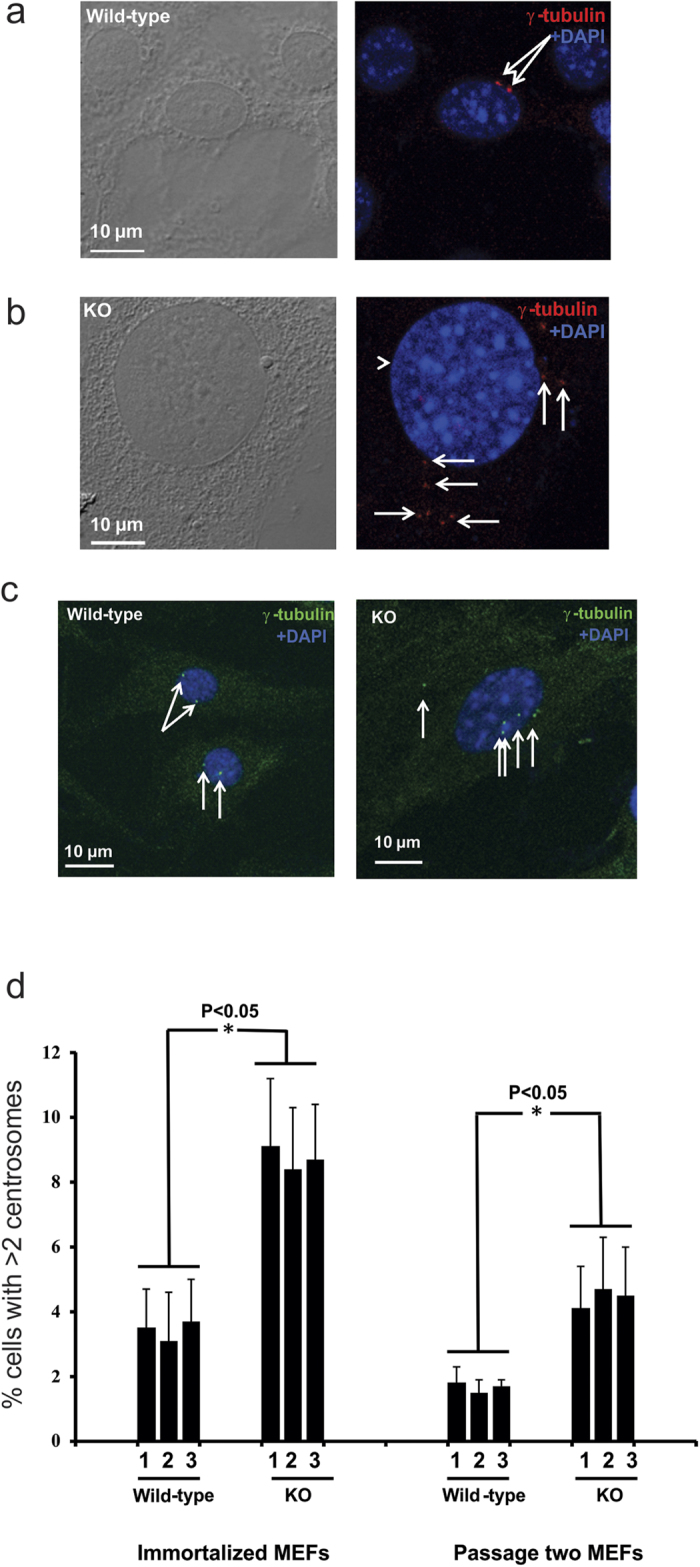
Multiple centrosomes in the *Spag6*-deficient (KO) MEFs. (**a**) Representative images of immortalized wild-type MEFs double stained with a monoclonal anti-γ tubulin antibody. Only two centrosomes were present in each wild-type MEFs. (**b**) Representative images of immortalized *Spag6*-deficient MEFs showing multiple centrosomes, and these centrosomes are not localized at the correct position. Frequently, abnormal large nuclei was seen in the *Spag6*-deficient MEFs (The arrow-heads point to abnormally large nuclei). (**c**) Representative images of passage two wild-type (left) and *Spag6*-deficient (right) MEFs stained with anti-γ tubulin antibody. Only two centrosomes were present in each wild-type MEF, but multiple centrosomes were observed in some *Spag6*-deficient MEFs. White arrows point to the centrosomes. (**d**) Quantitation of multiple centrosomes (>2) in primary and immortalized wild-type and *Spag6*-deficient MEFs. 500 cells were counted for each cell line, and three wild-type and three KO MEFs were analyzed. The percentages of cells with multiple centrosomes are significantly higher in the *Spag6*-deficient MEFs. *p < 0.05.

Some *Spag6*-deficient MEFs show large nuclei, we hypothesized that some cells might undergo abnormal cell division. The MEFs were stained with anti-γ-tubulin antibody to visualize centrosomes. In both immortalized and early passage number wild-type MEFs, multiple centrosomes were seen in very few cells (Fig. 5a and left panel of Fig. 5c). However, multiple centrosomes were frequently observed in both immortalized and early passage number *Spag6*-deficient MEFs (Fig. 5b and right panel of Fig. 5c), and the percentages of cells with multiple centrosomes were significantly higher in the *Spag6*-deficient MEFs (Fig. 5d), indicating a defect in cell division.

### Defective ciliogenesis in *Spag6*-deficient MEFs

**Figure 6:**
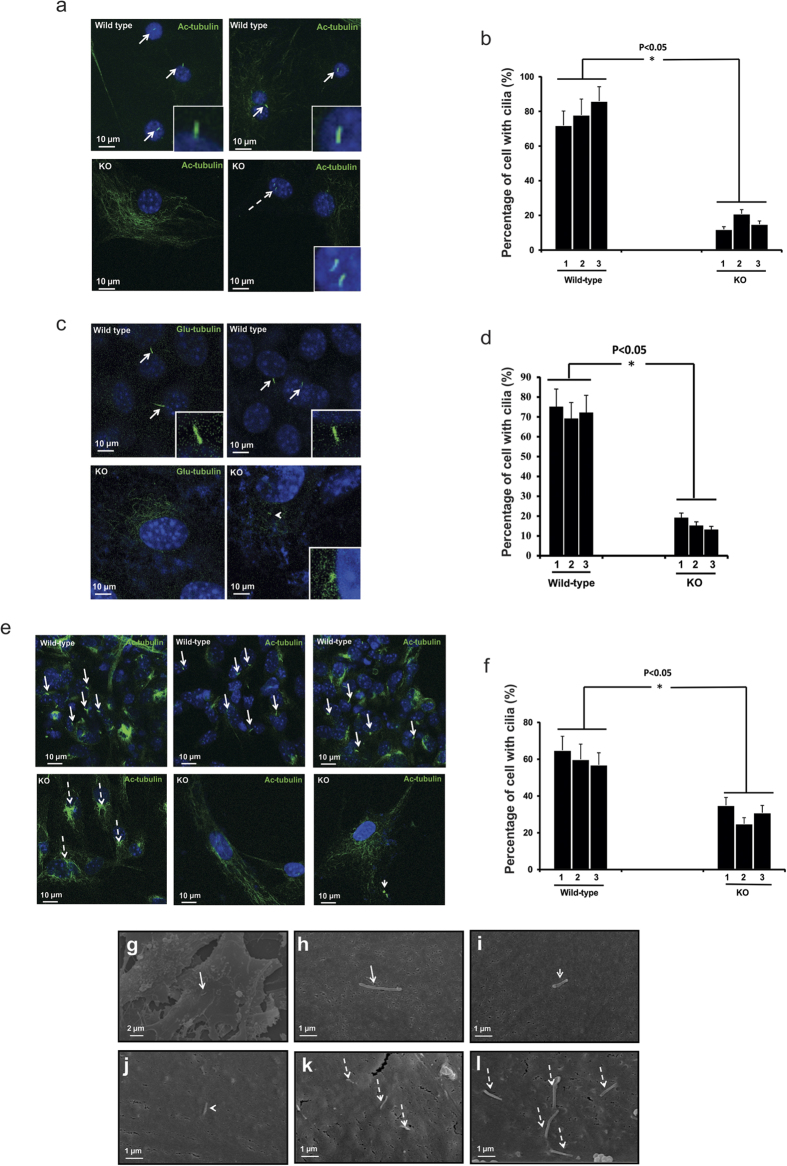
Ciliogenesis defects in the *Spag6*-deficient (KO) MEFs. After serum starvation, ciliogenesis in the immortalized (**a,c**) and passage two (**e**) wild-type and *Spag6*-deficient MEFs was examined by immunofluorescence labeling using an anti-acetylated tubulin, or an anti-Glu-tubulin antibody and SEM. (**a**) upper panel. Representative images of immortalized wild-type MEFs stained with acetylated tubulin antibody. Notice that most wild-type MEFs formed cilia, and each cell had only one cilium (arrows). The inserts show magnified images of cilia. (**a**) lower panel. Representative images of immortalized *Spag6*-deficient MEFs. Left panel shows a cell with acetylated tubulin signal in the cytoplasm, but no cilium was found. This labeling pattern is also shown in most *Spag6*-deficient MEFs. The right panel shows a cell with two cilia (dashed arrow). The insert is a higher magnification image of the two cilia. (**b**) The percentages of cells with cilia in the immortalized cells evaluated by anti-acetylated tubulin antibody. The percentage was significantly lower in the *Spag6*-deficient MEFs. Three wild-type and three *Spag6*-deficient MEF preparations were studied, and 500 cells were analyzed from each line. (**c**) The same experiment as a, except that an anti-Glu-tubulin antibody was used. The insert in the *Spag6* deficient panel shows a cell with a short cilium. (**d**) The percentages of cells with cilia in the immortalized cells evaluated with an anti-Glu-tubulin antibody. Notice that the percentages of cells with cilia are similar as evaluated by the anti-acetylated tubulin antibody. (**e**) Representative images of passage two wild-type (upper panel) and *Spag6*-deficient MEFs (lower panel). Most wild-type MEFs have cilia (arrows). However, very few *Spag6*-deficient MEFs have cilia (left two). The right panel shows a cell with a cilium (arrow head), but it is mis-localized. (**f**) The percentages of cells with cilia in the passage two cells. Similar to the immortalized cells, the percentage was significantly lower in the *Spag6*-deficient MEFs. Two wild-type and two *Spag6*-deficient MEF preparations were studied, and 500 cells were analyzed from each line. (**g**–**l**) Examination of cilia by SEM. (**g,h**) Representative images of wild-type MEFs at different magnification. Each wild-type cell has only one normal-looking cilium (arrows). (**i**–**l**): Representative images of *Spag6*-deficient MEFs with cilia. Most *Spag6*-deficient MEFs had no cilia (images not shown), but some cells still formed shorter (arrow heads in (**i,j**) and multiple cilia (dashed arrows in (**k,l**). *p < 0.05.

Cilia are formed from the mother centrioles. Given that abnormal centrosomes were observed in the *Spag6*-deficient MEFs, ciliogenesis was examined in these mutant cells. After serum starvation, immortalized and passage two wild-type and *Spag6*-deficient MEFs were stained with an anti-acetylated tubulin antibody and anti-detyrosinated alpha-tubulin (Glu-tubulin) antibody, which decorated cilia. Most wild-type MEFs developed cilia following serum deprivation and each cell formed only one cilium (Fig. 6a,c,e, upper panels). Given that a cilium is built from a mother centrosome, which is usually positioned close to the nucleus, the cilia in the wild-type MEFs are near the nuclei. However, cilia were found in fewer *Spag6*-deficient MEFs (Fig. 6a,c,e, lower panels). If present, they were commonly dislocated and distant from the nuclei (right lower panel in Fig. 6e). About 4% of *Spag6*-deficient cells analyzed showed multiple cilia (Fig. 6a, insert in the right lower panel). However, multiple cilia were not observed in the wild-type MEFs analyzed in our studies. In some *Spag6*-deficient MEFs, the acetylated or Glu-tubulin signal was observed in the cytoplasm, but not obvious in cilia (left lower panels in Fig. 6a,c, and left two panels in Fig 6e). Quantification of ciliogenesis in three pairs of wild-type and *Spag6*-deficient MEFs confirmed that the percentages of cilia-forming cells were significantly reduced in both immortalized and passage two *Spag6*-deficient MEFs as evaluated by both anti-acetylated tubulin and anti-Glu-tubulin antibodies (Fig. 6bdf).

Ciliogenesis in these MEFs was also examined by SEM. Consistently, wild-type MEFs had normal ciliogenesis with one cilium seen in each cell (Fig. 6gh). However, fewer *Spag6*-deficient MEFs formed cilia. If formed, they appeared to be shorter (Fig. 6ij), and some showed multiple cilia (Fig. 6k,l).

### SPAG6 specifically increases acetylated tubulin expression

**Figure 7:**
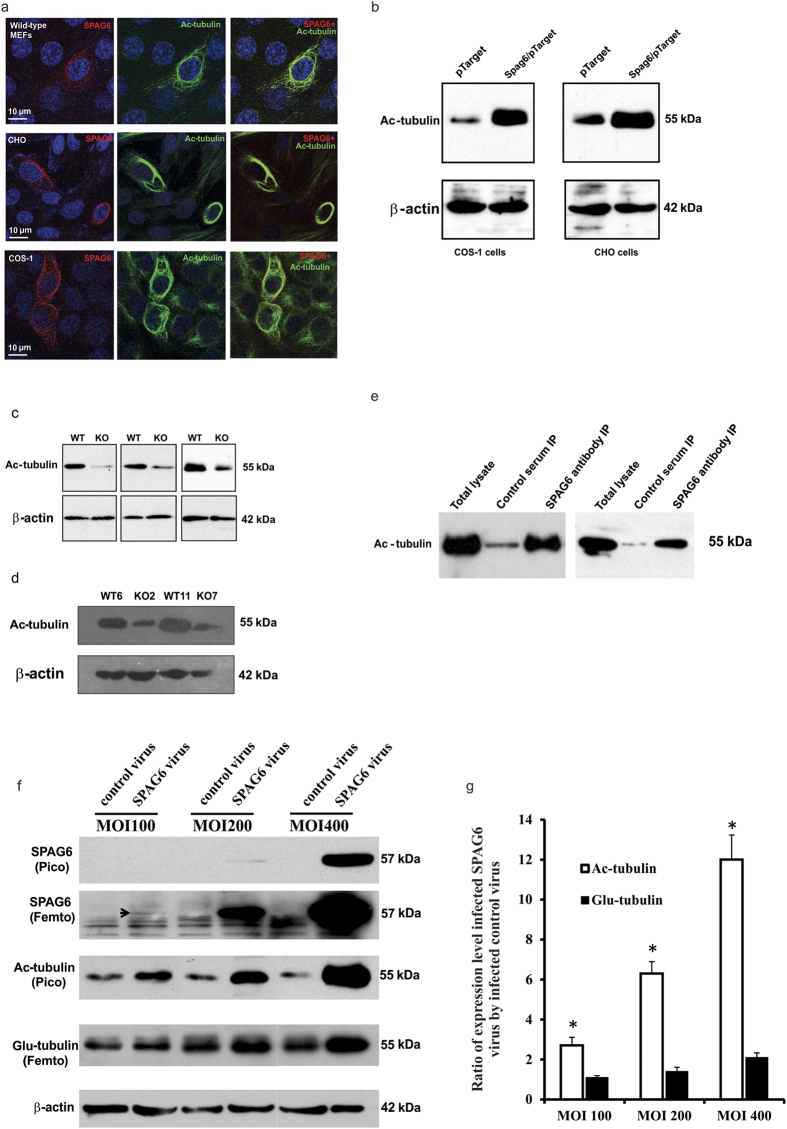
SPAG6 selectively stimulates acetylated tubulin expression in cultured cells. (**a**) Representative images showing SPAG6 co-localizes with acetylated tubulin in wild-type MEFs (upper panel), CHO cells (middle panel) and COS-1 cells (lower panel). (**b**) Overexpression of SPAG6 increases acetylated tubulin expression in COS-1 (left) and CHO (right) cells. Acetylated tubulin expression is dramatically reduced in the *Spag6*-deficient (KO) MEFs of both immortalized (**c**) and passage three (**d**) cells. Three independent pairs of MEFs were analyzed for the immortalized cells and two pairs for passage three cells. (**e**) Results from two independent co-IP experiments. Acetylated tubulin was co-pulled down by the anti-SPAG6 antibody from the COS-1 cells over-expressing SPAG6. (**f**) Comparison of expression of acetylated tubulin and Glu-tubulin in CHO cells infected with a *Spag6*-expressing virus. CHO cells were infected with increasing numbers of control and *Spag6*-expressing adenovirus particles, and expression levels of acetylated tubulin and Glu-tubulin were analyzed by Western blotting. Notice that even if there was a trace amount of SPAG6 expression when the cells were infected with MOI100 virus, the acetylated tubulin expression level was significantly increased. This increment was dramatically enhanced along with SPAG6 expression. However, there was no change in Glu-tubulin expression at MOI100. Even at MOI400, there was only a slight increase in Glu-tubulin expression. (**g**) Statistical analysis of expression of acetylated and Glu-tubulin. At all the three MOIs, increases in acetylated tubulin expression were significantly higher than Glu-tubulin. * p < 0.05.

To explore the molecular mechanism underlying the severe phenotypes caused by loss of *Spag6*, particularly the ciliogenesis defects, we overexpressed SPAG6 in mammalian cell lines. First, SPAG6/pTarget was transfected into wild-type MEFs. The transfected cells were double-labeled with a polyclonal anti-SPAG6 antibody and a monoclonal anti-acetylated tubulin antibody, which decorates cilia. Surprisingly, the cells overexpressing SPAG6 showed stronger staining for acetylated tubulin, and SPAG6 and acetylated tubulin completely co-localized (Fig. 7a, upper panel). In other mammalian cell lines such as COS-1 and CHO transfected with SPAG6/pTarget, we also observed a stronger signal for acetylated tubulin and that SPAG6 and acetylated tubulin were co-localized (Fig. 7a. Middle panel for CHO cells, and lower panel for COS-1 cells). Similar results were obtained when these cells were transfected with SPAG6/pcDNA3 plasmid to overexpress SPAG6 (data not shown).

The overexpression experiments suggest that SPAG6 is implicated in up-regulation of acetylated tubulin. To confirm this, we performed Western blot analysis for acetylated tubulin. As shown in Fig. 7b, overexpression of SPAG6 dramatically increased acetylated tubulin expression in COS-1 and CHO cells. Further, levels of acetylated tubulin in wild type MEFs were much higher than in both immortalized (Fig. 7c) and passage two (Fig. 7d) *Spag6*-deficient MEFs. Given co-localization of SPAG6 and acetylated tubulin, these two proteins might associate through physical interactions, co-immunoprecipitation experiments were conducted. COS-1 cells were transfected with SPAG6/pTarget plasmid, and the collected lysates were precipitated with an anti-SPAG6 polyclonal antibody or a control serum. Acetylated tubulin was pulled down only when the anti-SPAG6 antibody was used (Fig. 7e).

To determine if over-expression of SPAG6 also affects other types of tubulin post-translational modification, such as the formation of Glu-tubulin, an adenovirus expressing mouse SPAG6 with a GFP marker (Ade-GFP-SPAG6) was generated. To test SPAG6 expression, COS-1 and CHO cells were infected with the control virus and SPAG6-expressing virus, and Western blotting was carried out using the cell lysates. Cells infected with the control virus only expressed GFP. However, the cells infected with SPAG6-expressing virus expressed both GFP and SPAG6 (Supplemental Figure 2). CHO cells were infected with increasing titers of adenovirus, and cell lysates were analyzed expression levels of SPAG6, acetylated tubulin and Glu-tubulin. At Multiplicity of infection (MOI)100, there is a low level of SPAG6 expression when the cells were infected with Ad/SPAG6. The acetylated tubulin level was dramatically increased. However, there was no change in Glu-tubulin expression compared to the control. At MOI200, SPAG6 expression was significantly higher, and associated with higher acetylated tubulin expression and only a slight increase in Glu-tubulin expression compared to the control cells infected with the same titer of control adenovirus. At MOI400, SPAG6 expression was dramatically increased, and so was the acetylated tubulin expression with a modest increase in Glu-tubulin expression (Fig. 7f,g), indicating that the high level of SPAG6 not only increases acetylated tubulin expression, but also affects Glu-tubulin expression.

When CHO and wild-type MEFS were transfected with a SPAG6-expressing plasmid, and the cells were double stained with an anti-SPAG6 antibody and an anti-Glu-tubulin antibody, there was no increased Glu-tubulin expression in SPAG6-expressing cells (Supplemental Figure 3).

### Restoration of normal morphology and acetylated tubulin by forced expression of SPAG6 in *Spag6* null MEFs

**Figure 8:**
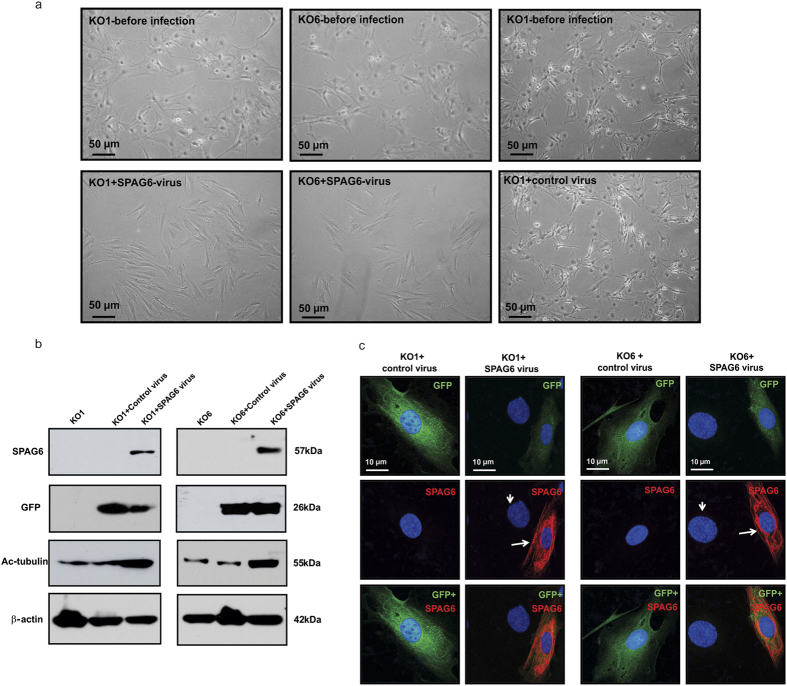
Forced-expression of SPAG6 rescues abnormal morphology observed in the *Spag6*-deficient (KO) MEFs. (**a**) Examination of cell morphology by light microscopy. Notice that before infection, the two *Spag6*-deficient MEFs had a large surface area. One day after infection, the cells treated with control virus maintained the same morphology (right panel). However, the cells infected with SPAG6-expressing virus showed reduced size, and changed to a fibroblast-like morphology. (**b**) Western blot analysis showed that once infected with the SPAG6-expression virus, the *Spag6*-deficient MEFs expressed both SPAG6 protein, and GFP, an indicator of virus infection. Expression of acetylated tubulin was also increased. The cells infected with control virus only expressed GFP, and the cells without any virus treatment expressed neither GFP nor SPAG6. (**c**) Immunofluorescence labeling images of the *Spag6-*deficient cells infected with control virus and SPAG6-expressing virus. Notice that the cells infected with control virus expressed GFP only, and the GFP positive area is larger than the cells expressing SPAG6, indicating a larger cell size. The cells expressing SPAG6 (arrows) have smaller nuclei than the non-SPAG6 expressing cells (arrow heads).

To determine if the phenotypes discovered in the *Spag6*-deficient MEFs could be rescued by re-expression of SPAG6, two independent *Spag6*-deficient MEF lines were infected with Ade-GFP-SPAG6 or control adenovirus (Ade-GFP). One day after infection, the cells infected with Ade-GFP-SPAG6 underwent a dramatic shape change. The cells shrank and reassumed the morphology of normal fibroblasts. These changes were not seen in the cells infected with Ade-GFP (Fig. 8a).

We next examined whether re-expression of SPAG6 also restored levels of acetylated tubulin in *Spag6*-deficient cells. Western blot analysis confirmed a strong increase in expression of acetylated tubulin in these cells re-expressing SPAG6 (Fig. 8b). Immunofluorescence labeling demonstrated that once infected with the *Spag6*-expressing adenovirus, the GFP-positive MEFs were smaller than the non-infected MEFs (Fig. 8c).

### Increased cell death rate of the *Spag6*-deficient MEFs in response to paclitaxel

**Figure 9:**
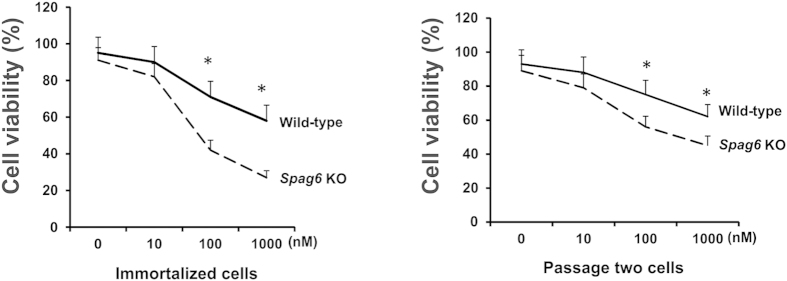
The *Spag6*-deficient (KO) MEFs are more sensitive to paclitaxel. Cell viability assay. Immortalized (left) and passage two (right) MEFs were treated with increasing concentrations of paclitaxel for 48 hr, and cell viability was measured. Notice that compared to the wild-type MEFs, viability is significantly lower in the *Spag6*-deficient MEFs when treated with 100 and 1000 nM of paclitaxel in both immortalized and passage two MEFs. *p < 0.05.

SPAG6 increases the expression of acetylated tubulin in the cells, suggesting that it affects microtubule stability. We next tested the response of both immortalized (left) and passage two (right) wild-type and *Spag6*-deficient MEFs to paclitaxel, a microtubule stabilizer, and a compound widely used for cancer therapy. After 48 hours treatment, cell viability was measured. In both immortalized and early passage MEFs, cell viabilities were significantly reduced in the *Spag6*-deficient MEFs (Fig. 9).

## Discussion

PF16 was originally cloned from *Chlamydomonas* as a cilia/flagella axonemal central apparatus protein, and a number of publications describe the role of *Spag6/PF16* in the regulation of cilia/flagella motility in other species^1^,^2^,^3^. Given the fact that all *Spag6*-deficient mice are smaller in size than their littermates, this phenotype cannot be fully explained by the function of a cilia/flagella motility regulator. Therefore, we hypothesized that mouse SPAG6 might have other functions.

Observations from *Spag6*-deficient MEFs supported this hypothesis. Without expression of the *Spag6* gene, MEFs underwent dramatic changes in cell morphology, cell growth, cell migration ability, cell adhesion, cell division and ciliogenesis. These changes suggested that mouse SPAG6 has additional functions beyond flagella/cilia motility regulation. These new findings are in line with a recent report that the mouse has two *Spag6* genes, a parental isoform, *Spag6*-BC061194, and an evolved gene from this parental isoform^24^, which we have been studying.

The mechanisms through which SPAG6 evolved to regulate diverse cellular functions are not known. Our previous investigations revealed that SPAG6 decorates microtubules^17^. *Spag6*-deficient MEFs have a ciliogenesis defect. We transfected wild-type and *Spag6*-deficient MEFs with a SPAG6 expression plasmid to determine if we could rescue the ciliogenesis abnormality. To our surprise, the *Spag6*-deficient MEFs resisted transfection. Even though the transfection efficiency was low, some wild-type MEFs overexpressed SPAG6. Surprisingly, these cells also overexpressed acetylated tubulin, and the two proteins co-localized near to the nuclei. To determine if this is a general phenomenon in mammalian cells, other cell lines, including COS-1, CHO cells were examined. In all of these cell lines, over-expression of SPAG6 was always associated with increased expression of acetylated tubulin, and the two proteins were always co-localized. Western blotting results demonstrated that the acetylated tubulin expression level was increased in these cells once they overexpressed SPAG6. In contrast, the acetylated tubulin expression level was dramatically reduced in the *Spag6*-deficient MEFs, and the reduced acetylated tubulin expression was rescued by forced-expression of SPAG6 using adenovirus infection.

Tubulin acetylation is one of the most important microtubule post-translational modifications^25^. Microtubules are dynamic polymers essential for cell morphogenesis, cell migration, cell division, and intracellular transport^26^,^27^,^28^,^29^. Acetylation of α-tubulin was first reported by L’Hernault and Rosenbaum^30^,^31^, who observed that α-tubulin in flagella was post-translationally modified. They mapped this modification to the ε-amino group of a single lysine residue. This process is mediated by tubulin acetyltransferase^32^. Several acetyltransferases have been identified including ARD1-NAT1^33^,^34^,^35^,^36^,^37^,^38^,^39^. A recent report demonstrated that αTAT1 is the major α-tubulin acetyltransferase in mice^40^. Increased expression of acetylated tubulin might also be due to decreased activity of deacetylases, such as histone deacetylases 6 (HDAC6)^41^ and SirT2^34^. It remains to be determined how SPAG6 promotes acetylated tubulin expression. One possibility is that SPAG6 regulates tubulin acetylation by affecting activity/protein expression of the known α-tubulin acetyltransferase or deacetylases. But we cannot rule out the possibility that SPAG6 regulates or influences the expression/activity of other tubulin acetylation factors.

Another possibility, which remains to be explored, is that SPAG6 itself functions as a tubulin acetyltransferase or an inhibitor of deacetylases. In *αTAT1* mutant mice, a loss of detectable K40 α-tubulin acetylation was observed in multiple tissues, but in some cellular structures such as cilia and axons, acetylation was normally enriched^40^. It is possible that SPAG6’s role in tubulin acetylation is cell type specific, or specifically in some cellular structures, such as in cilia.

Reduced acetylated tubulin expression levels in the *Spag6*-deficient MEFs could disrupt microtubule function, giving rise to the observed phenotypic changes in cell growth, migration, adhesion, division and ciliogenesis. This mechanism could also explain the increased number of cytoplasmic vesicles and the low transfection efficiency in the *Spag6*-deficient MEFs. Impaired microtubule-dependent vesicle transport could account for cytoplasmic vesicle accumulation, and impaired microtubule-mediated translocation of the lipid-DNA transfection complex into the nuclei could explain the extremely low transfection efficiency in the *Spag6*-deficient MEFs. The positive relationship of microtubule acetylation and transfection efficiency has been reported previously^42^.

To investigate if SPAG6 also regulates expression of other types of post-translational modification, Glu-tubulin expression was examined. Glu-tubulin, in which the C-terminal tyrosine of α-tubulin is removed by tubulin carboxypeptidase^43^, has a longer half time than tubulin. Overexpression of Glu-tubulin has been reported in malignant tumors^44^. Our results demonstrated that low expression levels of SPAG6 significantly increases acetylated tubulin expression, but not Glu-tubulin. However, when the SPAG6 expression level is increased, Glu-tubulin expression level was also modestly increased. These findings suggest that SPAG6 may regulate acetylated tubulin expression under physiological condition. Given the fact that SPAG6 is also up-regulated in cancers, high SPAG6 expression in these cancer cells may also increase Glu-tubulin expression.

In this study, we only highlighted the impact of alterations in SPAG6 on the α-tubulin/microtubule system. In the wound-healing experiment, the wild-type migrating cells showed a polarized F-actin distribution, but this pattern was not observed in the *Spag6*-deficient MEFs, suggesting that other cytoskeleton systems, such as actin filaments, might also be affected. It is known that conserved microtubule-actin interactions are essential for cell movement and morphogenesis^45^. It remains to be determined if SPAG6 has a direct role in the regulation of actin filaments, or indirectly through its influence on microtubules.

*Spag6*-deficient MEFs are very fragile. Even though the abnormal morphology and reduced acetylated tubulin levels were rescued by re-expression of SPAG6 using an adenovirus vector, almost all the cells died three to four days after virus infection. SPAG6 may quickly restore the microtubule/cytoskeleton system in the *Spag6*-deficient MEFs, but the mutant cells cannot accommodate to the sudden environmental change. This phenotype was not observed in the wild-type MEFs infected with SPAG6-expressing virus or the *Spag6*-deficent MEFs infected with the control virus. Due to the short life span of the rescued MEFs, we were not able to determine if cell growth, migration, division, and ciliogenesis also recover. The fragility of the *Spag6* virus-infected cells may reflect a time or dose-dependence of SPAG6 action such that perfect titration of expression must be achieved to support normal cellular function.

It is not surprising that SPAG6, previously known as a motile cilia/flagella central apparatus protein, has additional functions. It is a microtubule-associated protein, but is only assembled into a cilium during ciliogenesis. However, before cilia are assembled, the protein is already expressed. The full length protein has multiple contiguous armadillo repeats, a conserved domain for protein-protein interaction^46^. Consequently, SPAG6 might associate with multiple proteins in different cell types, or even with different proteins at different cell cycle stages in the same cell. Given its tubulin binding ability, the primary role of SPAG6 appears to lie in the regulation of the microtubule/cytoskeleton system.

It has been reported that human *SPAG6* expression is increased in several human malignancies^8^,^9^,^10^. We speculate that overexpression of SPAG6 might be the reason why some cancers are resistant to microtubule-targeting drugs, such as paclitaxel. The fact that the *Spag6*-deficient MEFs are more sensitive to paclitaxel suggests that reducing SPAG6 expression in paclitaxel-insensitive cancers might be an effective therapeutic approach.

## Additional Information

**How to cite this article**: Li, W. *et al.* Sperm Associated Antigen 6 (SPAG6) Regulates Fibroblast Cell Growth, Morphology, Migration and Ciliogenesis. *Sci. Rep.* **5**, 16506; doi: 10.1038/srep16506 (2015).

## Supplementary Material

Supplementary Information

Supplementary movie 1

Supplementary movie 2
