# Supplementary material for: Sperm Associated Antigen 6 (SPAG6) Regulates Fibroblast Cell Growth, Morphology, Migration and Ciliogenesis
Source: Sci Rep. 2015 Nov 20;5:16506. doi: 10.1038/srep16506 (PMC4653743; doi:10.1038/srep16506)
Supplement: Supplementary Information [file srep16506-s1.pdf]

Sperm Associated Antigen 6 (SPAG6) Regulates Fibroblast Cell Growth, Morphology, Migration and Ciliogenesis

Wei Li[1], Abir Mukherjee[2], Jinhua Wu[1], Ling Zhang[1, 3], Maria E Teves[1], Hongfei Li[1], Shanti Nambiar[1], Scott C Henderson[4], Alan R Horwitz[5], Jerome F Strauss III[1, 2], Xianjun Fang[2], Zhibing Zhang[1, 2]

1. Department of Obstetrics & Gynecology, 2. Department of Biochemistry and Molecular Biology, Virginia Commonwealth University, Richmond, VA, 23298; 3. School of Public Health, Wuhan University of Science and Technology, Wuhan, Hubei, 430081, China. 4. Department of Anatomy and Neurobiology, Virginia Commonwealth University, Richmond, VA, 23298, 5. Department of Cell Biology, University of Virginia, Charlottesville, VA 22908, USA.

Running title: SPAG6, Acetylated Tubulin and Cell Function

Address correspondence to: Zhibing Zhang, MD, PhD

Associate Professor

Department of Obstetrics/Gynecology

Virginia Commonwealth University

Richmond, VA, 23298

Email: zzhang4@vcu.edu

Supplemental information.

Supplemental Figure 1. Analyses of cell morphology by scan EM and Crystal blue staining.

Morphology of the wild-type and *Spag6*-deficient MEFs was analyzed by SEM (left panel) and crystal blue staining (right panel). Notice that *Spag6*-deficient MEFs have larger cell surface.

Supplemental Figure 2. Examination of SPAG6 protein expression by Western blot analysis using the COS-1 and CHO cells infected with adenovirus.

The COS-1 and CHO cells were infected with control adenovirus virus or the adenovirus expressing SPAG6. GFP is expressed in the cells infected with either control virus or SPAG6-expressing virus. SPAG6 is only expressed when the SPAG6-exprtessing adenovirus was used.

Supplemental Figure 3. Exogenous expression of SPAG6 does not increase Glu-tubulin expression in MEFs and CHO cells.

Wild-type MEFs (a) and CHO cells (b) were transfected with SPAG6/pTarget. Two days after transfection, the cells were double stained with an anti-SPAG6 polyclonal antibody and an anti-Glu-tubulin antibody. Notice that there was no increased Glu-tubulin (in green) expression in the cells expressing SPAG6 (in red).

Supplemental movie 1.

Representative movie of wound-healing assay for wild-type MEFs. Notice that the cells migrated very fast, and the wound area was occupied in about six hours after scratch.

Supplemental movie 2.

Representative movie of wound-healing assay for *Spag6*-deficient MEFs. Notice that the cells migrated slowly, and the wound area was still open even six hours after scratch.

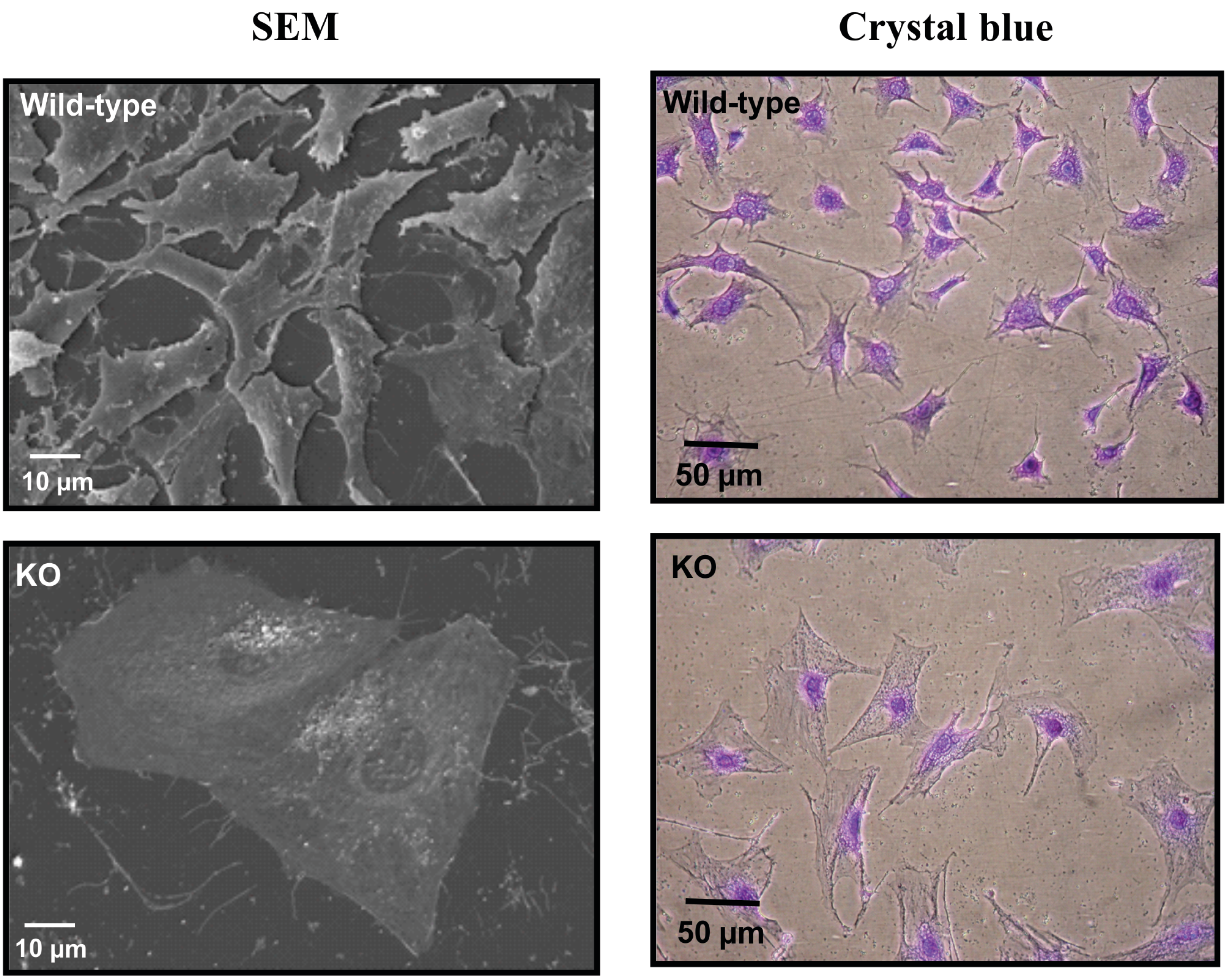
SEM
Crystal blue
Wild-type
10 µm
Wild-type
50 µm
KO
10 µm
KO
50 µm

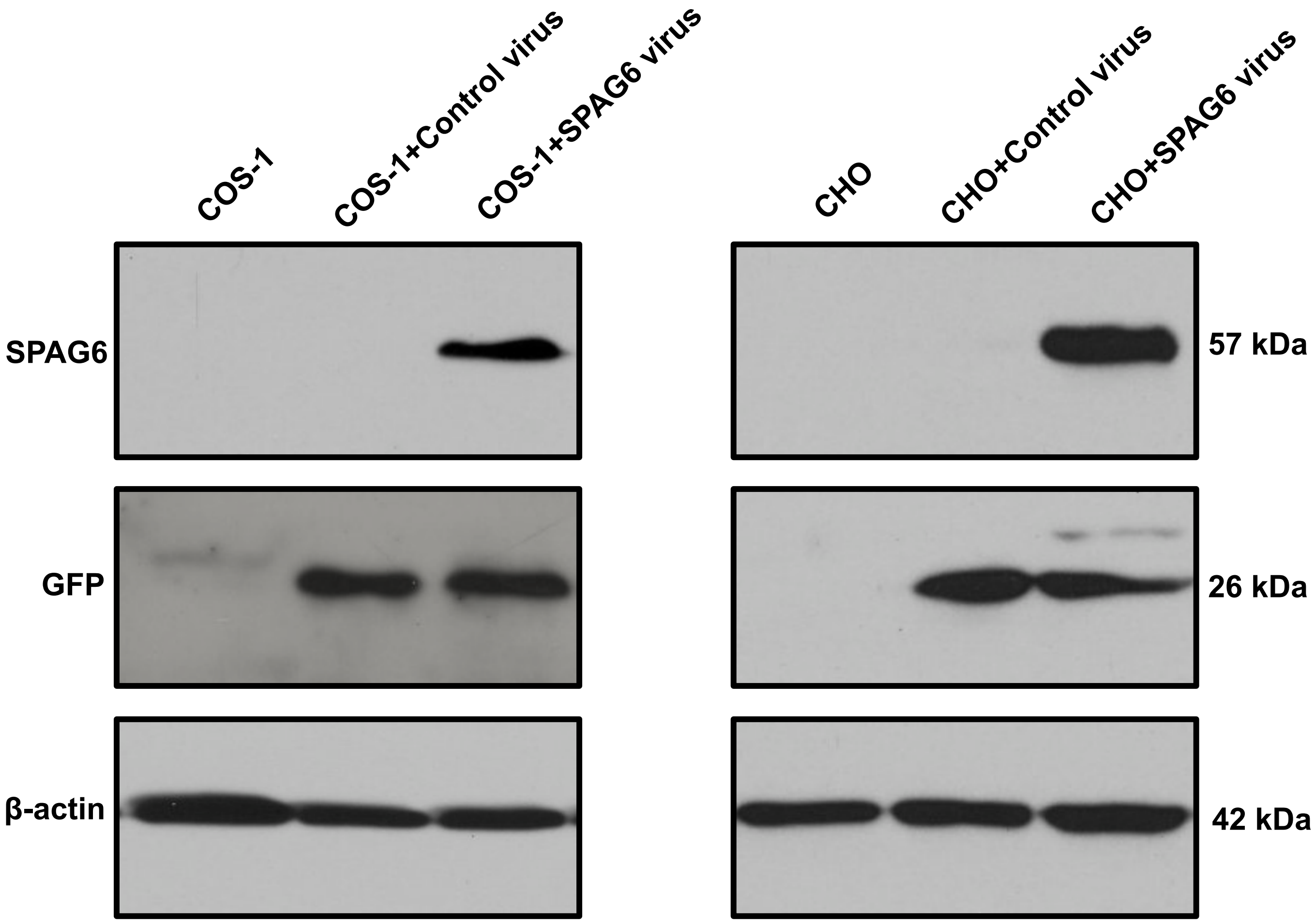
COS-1
COS-1+Control virus
COS-1+SPAG6 virus
CHO
CHO+Control virus
CHO+SPAG6 virus
SPAG6
GFP
β-actin
57 kDa
26 kDa
42 kDa

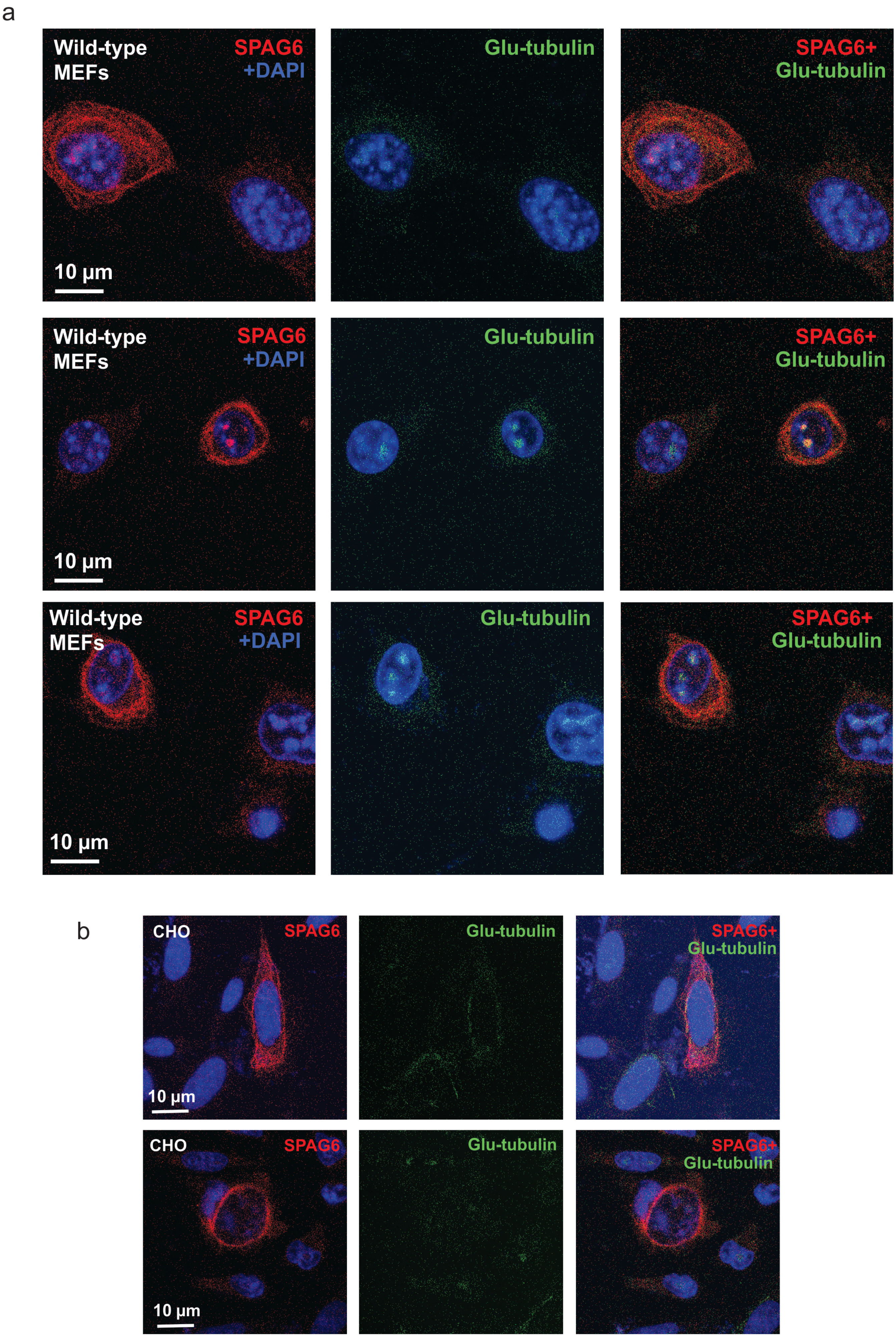
a
Wild-type MEFs
SPAG6 +DAPI
Glu-tubulin
SPAG6+ Glu-tubulin
10 µm
b
CHO
SPAG6
Glu-tubulin
SPAG6+ Glu-tubulin
10 µm
